# Supplementary material for: Detecting topological variations of DNA at single-molecule level
Source: Nat Commun. 2019 Jan 2;10:3. doi: 10.1038/s41467-018-07924-1 (PMC6315031; doi:10.1038/s41467-018-07924-1)
Supplement: Supplementary file 1 — Supplementary Information [file 41467_2018_7924_MOESM1_ESM.pdf]

Supplementary information.

“Detecting topological variations of DNA at single-molecule level.”

Ke Liu, Chao Pan, Alexandre Kuhn, Adrian Pascal Nievergelt, Georg E. Fantner, Olgica Milenkovic, and Aleksandra Radenovic

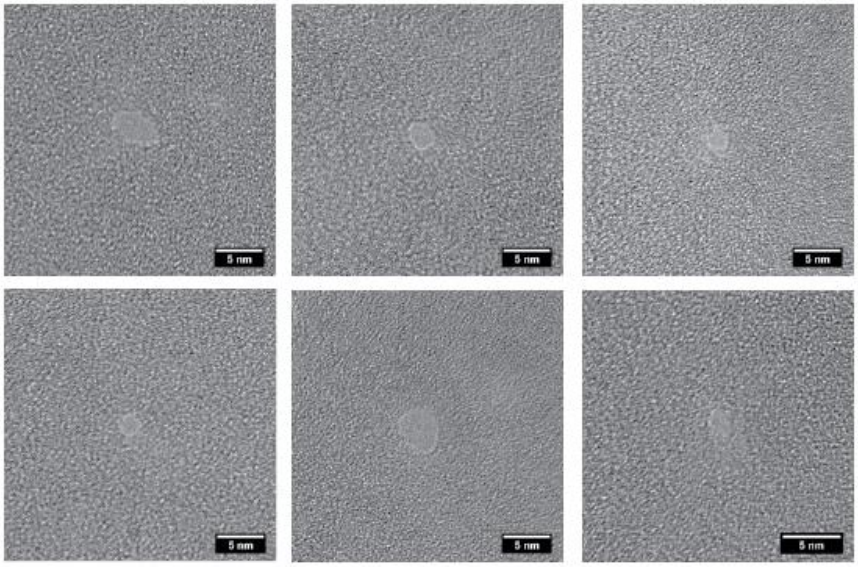

Supplementary Figure 1. TEM images of nanopores drilled by focused electron beam.

Supplementary Discussion 1: Conductance models.

The discrete levels of individual events were used to calculate the pore size and the pore length using a widely-accepted conductance model.[1, 2]

For the circular nanopores, G_{open} can be calculated by the equation:

$$G_{open} = \sigma_{bulk}\left[\frac{4L}{d^2\pi} + \frac{1}{d}\right]^{-1} \quad (1)$$

When the DNA segment is inside the nanopore, the effective nanopore diameter d_{eff} will be

$$d_{eff} = \sqrt{d^2 - d_{DNA}^2} \quad (2)$$

In above case, ss segment and ds segment can be 1.5 nm and 2.2 nm in diameter, respectively.

Therefore, we can determine G_{bloc} to be

$$G_{bloc} = \sigma_{bulk}\left[\frac{4L}{d_{eff}^2\pi} + \frac{1}{d_{eff}}\right]^{-1} \quad (3)$$

As a result, we can compute the pore length (L) and the pore diameter (d) according to experimentally measured G_{open} and G_{bloc}.

	Open pore current (nA)	Current blockade (nA)	Analyte diameter (nm)	Computed pore size (nm)	Computed pore length (nm)
ssDNA	4.2	1.2	1.5	2.7	8.8
dsDNA	4.2	2.4	2.2	2.8	9.5

Supplementary Table 1. Computed nanopore parameters.

Supplementary Discussion 2: Noise models.

1. The Anderson-Darling Test

The Anderson-Darling test[3] is a commonly used parameter-free method for determining whether a set of data points is drawn from a given distribution (e.g., Gaussian). The test statistic belongs to the family of quadratic empirical distribution function statistics, which measure the distance between the hypothesized distribution, F(x) and the empirical cumulative distribution function, $F_n(x)$, according to:

$$\mathrm{D} = \mathrm{n}\int_{-\infty}^{\infty}\left(F_n(x) - F(x)\right)^2 \omega(x)\, dF(x) \qquad (4).$$

Here, n stands for the number of data points, while w(x) the weight function equals

$$\omega(x) = \left[F(x)\left(1 - F(x)\right)\right]^{-1} \qquad (5).$$

It is obvious that the Anderson-Darling test places greater weight on the observations from the tails of the distribution. The Anderson-Darling test statistic reads as

$$A_n^2 = -\mathrm{n} - \sum_{i=1}^{n}\frac{n}{2i-1}\left[\ln\left(F(x_i)\right) + \ln\left(1 - F(x_{n+1-i})\right)\right] \qquad (6).$$

It is implemented in MATLAB as part of the command *adtest*, and the decision to reject or not reject the null hypothesis is based on comparing the p-value for the hypothesis test with the specified significance level, and not on comparing the test statistic with a chosen critical value.

2. Gaussian Mixture Models (GMM)

A Gaussian distribution N $(x|\mu, \sigma^2)$ with mean μ and variance σ^2 in one dimension has the familiar probability density function

$$p_N(x) = \frac{1}{\sqrt{2\pi}\sigma}e^{-\frac{(x-\mu)^2}{2\sigma^2}} \qquad (7).$$

The probability distribution of a GMM with K components may be written as

$$\mathrm{p}(x) = \sum_{k=1}^{K} p(z=k)p(x|z=k) = \sum_{k=1}^{K}\pi_k p_{N,k}(x) \qquad (8),$$

where z is a latent variable indicating which component in the mixture a point x belongs to, while $\mathrm{p}_N, k(x)$is a Gaussian distribution with mean μ_k and variance ρ_k^2; π_k is the weight of k-th component, and clearly

$$\sum_{k=1}^{K}\pi_k = 1 \qquad (9).$$

The parameters in a GMM model are estimated using the maximize likelihood method and accompanying E-M algorithm.[4]

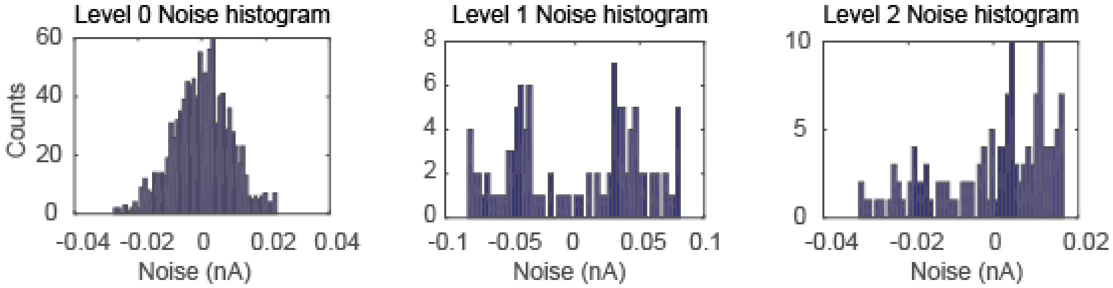

Supplementary Figure 2. Noise histograms for different levels.

3. Welch's Method for Estimating PSDs

Welch's method is based on the concept of using periodogram spectrum estimates, which are the result of transforming a signal from the time domain to the frequency domain. The main component of the method is the Fast Fourier Transform (FFT). The method involves two preprocessing steps: first, the signal is split up into overlapping segments; second, each segment is windowed. In the first step, the length of the overlapping fragments can be arbitrary (in the range (0,100)%), and the length of each segment is a tunable parameter. For the second step, commonly used windows includes rectangular, Hanning and Blackman-Harris windows. The squared magnitude of the periodogram is computed using the FFT after preprocessing each segment individually. The results are averaged to reduce the variance of individual power measurements. In our analysis, we choose the length of the segment to be N/32, where N is the total length of the signal, and do not use any overlaps. For windowing, we chose the Blackman-Harris window.

It is implemented in MATLAB as part of the command *pwelch*. The following formula in used in the command *pwelch* to convert $P_o[i]$ into a real signal power $P_{sig}[i]$ at frequency index i:

$$P_{sig}[i] = P_0[i] \cdot \frac{NG \cdot f_{bin}}{CG^2} \qquad (10),$$

where f_{bin} is the frequency resolution, and NG and CG are two variables related to the choice of windowing.

4. Hilbert-Huang Transform

The Hilbert–Huang Transform (HHT) is a transform designed for empirical analyses of nonlinear and non-stationary data. The transform, being unconstrained by the Heisenberg principle, can lead to high resolution in both the frequency and time domains. It relies on two processing tools: the Hilbert spectral analyzer (HAS) and the empirical mode decomposition (EMD) framework. The first step in the analysis is to find intrinsic mode functions (IMF) of the signal then apply the HAS to obtain instantaneous frequency data.

4.1 Empirical mode decomposition (EMD)

To simplify data analysis, the signal is decomposed into a finite and small number of components using EMD technique. An algorithmic depiction of the process is presented in the previous publication.[5]

4.2 The Hilbert transform

The Hilbert transform of a signal $\mathrm{x(t)}$ is defined as:

$$\mathrm{y(t)} = \frac{1}{\pi}\int_{-\infty}^{\infty}\frac{x(\tau)}{t-\tau}d\tau \qquad (11).$$

By applying the Hilbert transform to every IMF component $\mathrm{x_j(t)}$, we obtain a representation that allows us to extract local properties of the signal. The process works as follows: After obtaining all IMFs, the original signal $\mathrm{x(t)}$ can be written as:

$$x(t) = r(t) + \sum_{j=1}^{k} x_j(t) \qquad (12),$$

where $\mathrm{r(t)}$ is the residual, k is the number of intrinsic mode functions, $\mathrm{x_j(t)}$ is the j-th IMF. Let $\mathrm{y_j(t)}$ denotes the Hilbert transform of $\mathrm{x_j(t)}$. Each $\mathrm{x_j(t)}$ in turn has a real and an imaginary component that reads as:

$$x_j(t) = Real(a_j(t)e^{-i\varphi_j(t)}), y_j(t) = Im(a_j(t)e^{-i\varphi_j(t)}) \qquad (13).$$

The instantaneous frequency is defined as $\mathrm{\omega_j(t)} = \frac{d\varphi_j(t)}{dt}$, while the Hilbert spectrum of $\mathrm{x_j(t)}$ is defined as

$$H_j(\omega, t) = \begin{cases} a_j(t), & \omega = \omega_j(t) \\ 0, & otherwise \end{cases} \qquad (14).$$

The estimated Hilbert Spectrum of the signal itself $\mathrm{x(t)}$ equals:

$$H(\omega, t) = \sum_{j=1}^{k} H_j(\omega, t) \qquad (15).$$

This representation can be used to analyze the characteristics of instantaneous frequencies for non-stationary signals.

We performed the HHT transform and Hilbert spectral analysis on our “212” readouts. The results are summarized below. Figures S3 plot the components of the EMD for one sample of 3 level signals. Different samples follow the same trend. The HHT results depicted in Figures S4 show that in the HHT domain, Level 1 signals tend to have larger energy in the given frequency range than Level 0 and 2 signals, so that energy may be consequently used as a means to discriminate them. Better results using HHT may be expected for a larger number of events, as our analysis only made use of 40 samples for each level.

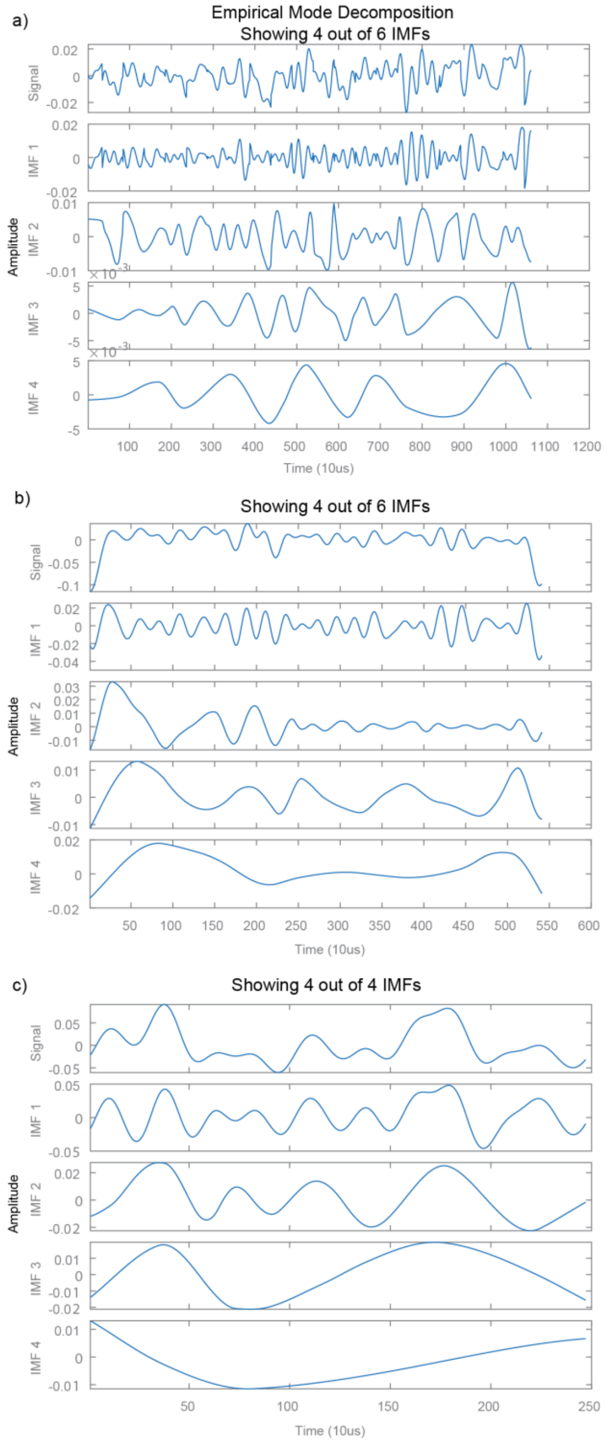

Supplementary Figure 3. EMD of Level 0, 1 and 2 event. (a) for Level 0, (b) for Level 1 and (c) for Level 2.

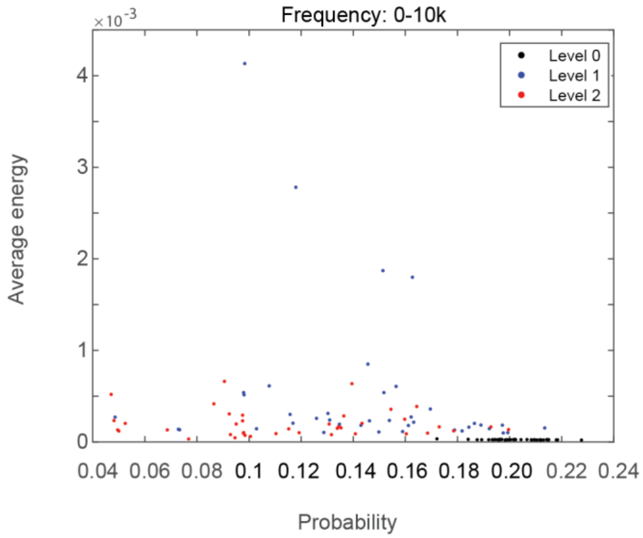

Supplementary Figure 4. A typical example of average energy vs. probability with HHT of Level 0, 1 and 2 signals in the frequency domain for 0-10k Hz.

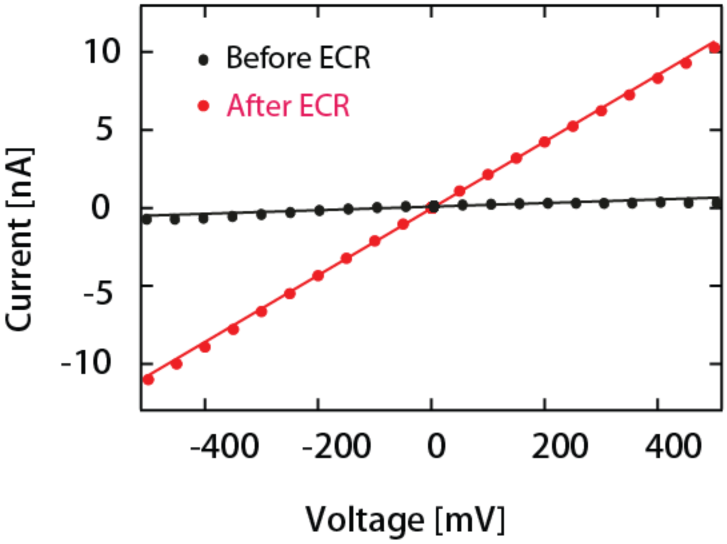

Supplementary Figure 5. Current-voltage characteristics of a MoS_2 membrane before and after ECR process.

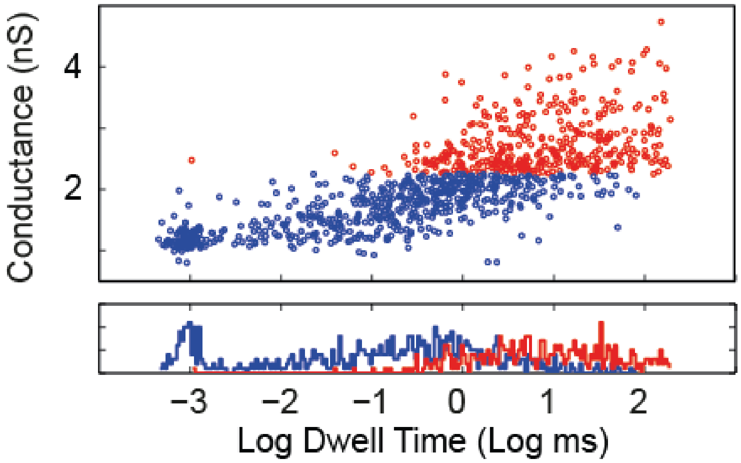

Supplementary Figure 6. Scatter plot of translocation events of protruded complex (red) and ssDNA (blue).

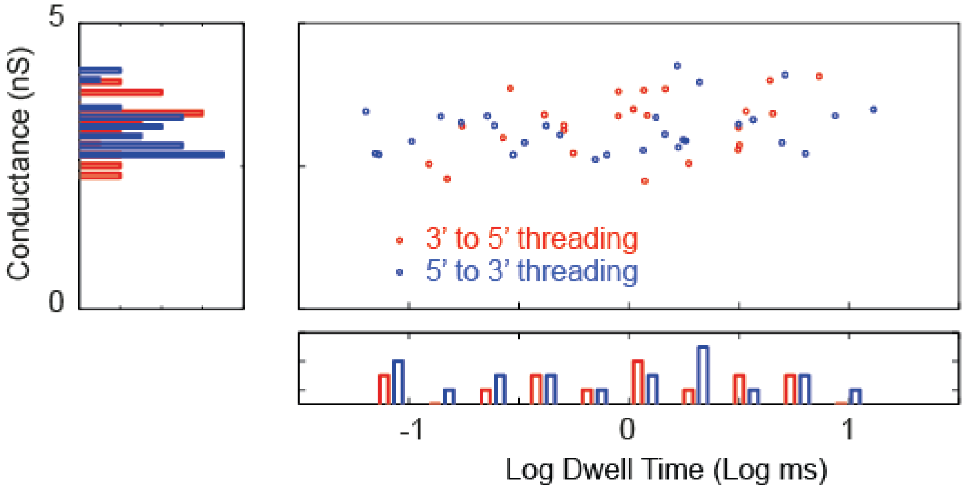

Supplementary Figure 7. Scatter plot of 3' end entry and 5' end entry.

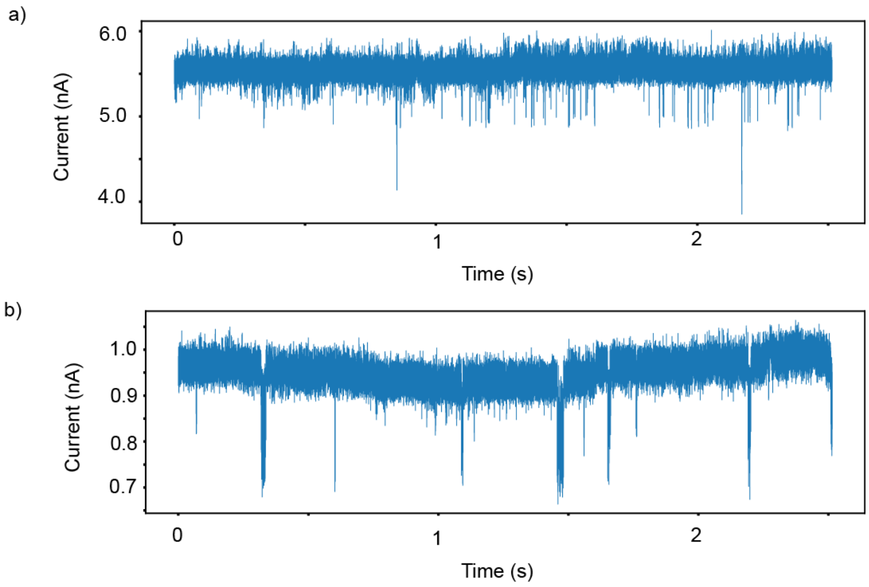

Supplementary Figure 8. Representative continuous traces for a) SiN_x nanopore recorded at 200 mV and room temperature using ss-ds-ss DNA complexes in 4M LiCl and for b) MoS_2 nanopore recorded at 200 mV and 4°C using barcoded short 22mer in 4M LiCl.

Supplementary References

1. Wanunu, M. et al. Rapid electronic detection of probe-specific microRNAs using thin nanopore sensors. *Nat. Nanotechnol.* **5**, 807-814 (2010).
2. Kowalczyk, S.W., Grosberg, A.Y., Rabin, Y. & Dekker, C. Modeling the conductance and DNA blockade of solid-state nanopores. *Nanotechnology* **22**, 315101 (2011).
3. Anderson, T.W. & Darling, D.A. Asymptotic Theory of Certain Goodness of Fit Criteria Based on Stochastic Processes. *Ann Math Stat* **23**, 193-212 (1952).
4. Redner, R.A. & Walker, H.F. Mixture Densities, Maximum-Likelihood and the Em Algorithm. *Siam Rev* **26**, 195-237 (1984).
5. Jain, M. et al. Nanopore sequencing and assembly of a human genome with ultra-long reads. *Nat. Biotechnol.* **36**, 338-345 (2018).
