# Supplementary material for: Detecting topological variations of DNA at single-molecule level
Source: Nat Commun. 2019 Jan 2;10:3. doi: 10.1038/s41467-018-07924-1 (PMC6315031; doi:10.1038/s41467-018-07924-1)
Supplement: Supplementary file 3 — Description of Additional Supplementary Files [file 41467_2018_7924_MOESM3_ESM.pdf]

Title: Supplementary Software

Description: Contains all code used in the analysis of the data presented in the manuscript.
